# *ABCB1* 3435TT and *ABCG2* 421CC genotypes were significantly associated with longer progression-free survival in Chinese breast cancer patients

**DOI:** 10.18632/oncotarget.22201

**Published:** 2017-10-31

**Authors:** Wanjun Li, Dan Zhang, Fen Du, Xuemei Xing, Ying Wu, Dong Xiao, Ming Liang, Zhigang Fan, Peng Zhao, Tao Liu, Guoyin Li

**Affiliations:** ^1^ Department of Pathology, Hanzhong 3201 Hospital Affiliated to Xi’an Jiaotong University, Xi’an, Shaanxi, China; ^2^ Department of Oncology, Hanzhong 3201 Hospital Affiliated to Xi’an Jiaotong University, Xi’an, Shaanxi, China; ^3^ Department of Nursing, Hanzhong Vocational Technical College, Hanzhong, Shaanxi, China; ^4^ Department of Clinical Laboratory, Hanzhong 3201 Hospital Affiliated to Xi’an Jiaotong University, Xi’an, Shaanxi, China; ^5^ Department of Breast and Vascular Surgery, Xijing Hospital, The Fourth Military Medical University, Xi’an, Shaanxi, China; ^6^ Department of General surgery, Hanzhong 3201 Hospital Affiliated to Xi’an Jiaotong University, Xi’an, Shaanxi, China; ^7^ Department of Hematology, Hanzhong 3201 Hospital Affiliated to Xi’an Jiaotong University, Xi’an, Shaanxi, China; ^8^ Department of Ophthalmology, Hanzhong 3201 Hospital Affiliated to Xi’an Jiaotong University, Xi’an, Shaanxi, China; ^9^ Department of Biochemistry and Molecular Biology, The Fourth Military Medical University, Xi’an, Shaanxi, China

**Keywords:** breast cancer, *ABCB*1 C3435T and *ABCG*2 C421A, single nucleotide polymorphism, mutation rate, progression-free survival

## Abstract

**Objective:**

To investigate the distribution of *ABCB1* C3435T and *ABCG2* C421A gene polymorphisms in Chinese Han population and their influences on the susceptibility and prognosis of breast carcinoma.

**Methods:**

A total of 200 female subjects were enrolled in this study, comprising 100 breast cancer patients and 100 healthy controls. Carcinoma and para-carcinoma tissues were collected from the breast cancer patients, while peripheral blood was collected from healthy controls. Single nucleotide polymorphisms (SNPs) were detected by the Taqman method. Progression-free survival (PFS) and 5-year survival rate of the patients were calculated.

**Results:**

*ABCB*1 C3435T and *ABCG*2 C421A polymorphisms were not associated with disease susceptibility and 5-year survival rate in the study population (*p*>0.05). However, a high mutation rate of both *ABCB*1 C3435T and *ABCG*2 C421A (16% and 17%, respectively) was observed in breast cancer tissues. Patients with *ABCB*1 3435TT genotype or *ABCG*2 421CC genotype had longer PFS (*p*<0.05).

**Conclusion:**

*ABCB*1 3435TT and *ABCG*2 421CC were significantly associated with longer PFS in Chinese breast cancer patients.

## INTRODUCTION

Breast carcinoma is the leading cause of cancer-related death among female patients with malignant tumors worldwide [1]. The morbidity and mortality rates of breast carcinoma have steadily increased since 1980s [2]. In the past few decades, great progress has been made in the treatment of breast cancer, particularly in the field of chemotherapy. However, treatment efficacy varies greatly due to the inherent genetic heterogeneity of individuals.

Efflux of agents from oncocyte by ATP-binding cassette (ABC) transporter is the most predominant and common mechanism of multiple drug resistance (MDR) among carcinoma [3]. In humans, 49 ABC genes have been reported and classified into seven different families [4, 5]. However, ABC gene subtypes involved in drug efflux from human cells do not belong to any particular family. For example, 12 transporters have been reported to regulate drug efflux; however, permeability glycoprotein (P-gp/ABCB1), multidrug resistance protein (MRP1/ABCC1), and breast cancer resistance protein (BCRP/ABCG2) are important for the efflux of a variety of drugs [3].

Human *ABCB*1 gene is located in chromosome region 7q21; this gene encodes a transmembrane transporters of 170 kDa that acts as an efflux pump for a variety of environmental carcinogens and antineoplastic drugs and plays an important role in resulting MDR of tumors [6-9]. Previous studies have shown that 66 coding single-nucleotide polymorphisms (SNPs) in *ABCB*1 gene have been identified, including 22 synonymous mutations and 44 non-synonymous mutations [10]. Several studies suggested that the expression level of *ABCB*1 gene was influenced by its polymorphism status [11, 12]. One of the most critical *ABCB*1 gene polymorphism is C3435T (rs1045642). Although it is a synonymous mutation, C3435T can alter the mRNA expression level of *ABCB*1, protein activity, and substrate specificity [13-15]. The variant allele frequency of C3435T is significantly different among various populations and races [16].

Human *ABCG*2 gene is located in chromosomal region 4q22 and encodes a 72-kDa membrane protein [17]. ABCG2 can transport numerous substrates, ranging from chemotherapeutics to carcinogenic xenobiotics [18-20]. Thus far, several SNPs in *ABCG*2 gene have been identified that can alter its expression and functionality [21]. One of the most important *ABCG*2 gene polymorphisms is C421A (rs2231142). The C421A SNP can lead to a glutamine-to-lysine amino acid substitution, resulting in decreased expression and activity of the ABCG2 protein [22-24]. Similar to C3435T, the mutation rate of C421A in *ABCG*2 gene is significantly different among different populations [25].

This study investigated the distribution of rs1045642 and rs2231142 polymorphisms in a Chinese Han breast cancer population who had been treated with post-operative chemotherapy. Correlation between the genetic polymorphism and breast cancer incidence, clinical features, and prognosis were explored.

## RESULTS

### Baseline characteristics of study subjects

The distributions of characteristics of the 100 breast cancer cases and 100 healthy controls are presented in Table 1. There were no significant differences in the distributions of age and menopausal state between cases and controls (*p*=0.571 and *p*= 0.48, respectively), and the average age was matched for breast cancer cases (range, 23–77 years; median, 50 years) and controls (range, 20–75 years; median, 50 years). Of the 100 breast cancer cases, 75 had invasive ductal carcinoma, 20 had invasive lobular carcinoma, and 5 had medullary carcinoma. Furthermore, 63 patients were diagnosed with stage II and 37 patients were diagnosed with stage III. Age at menarche of the patients was 12-18 years old. None of them had family history of breast cancer. Of the patients in our cohort, 70% / 60% / 40% were estrogen receptor (ER) /progesterone receptor (PR) / Her2 positive, respectively. IHC results showed 34% / 38% / 28% of patients with low / intermediate / high Ki67 expression. Lymph node metastasis was detected in 52% of the patients.

**Table 1:** Demographic and clinicopathological parameters of patients (n = 100)

Characteristic	Case number	Controls number	*P*
Age (years)			
Median	50	50	
Range	23-77	20-75	
<50	44	50	0.479
≥50	56	50	
Age at menarche (years)			
≤14	68		
>14	32		
Menopausal state			
Premenopausal	48	54	0.48
Postmenopausal	52	46	
Reproductive history			
One child	35		
Two children	53		
Three or more children	12		
Onset age (year,¯x±s)	51.29±9.48		
Family history			
Yes	0		
No	100		
Five year survival rate	65		
Pathological location			
Left breast	53		
Right breast	47		
Pathological type			
Invasive ductal carcinoma	75		
Invasive lobular carcinoma	20		
Medullary carcinoma	5		
Clinical stage			
II	63		
III	37		
Estrogen receptor			
+	70		
-	30		
Progesterone receptor			
+	60		
-	40		
Her2			
+	40		
-	60		
Ki67			
Low (<14%)	34		
Intermediate (14%-30%)	38		
High (>30%)	28		
Lymph node metastasis			
Node-positive	52		
Node-negative	48		
Surgery			
Yes	100		
No	0		
Postoperative chemotherapy			
Yes	96		
No	4		

### Comparison of genotype distribution between patients with breast carcinoma and healthy controls

Overall genotype and allele frequencies for *ABCB*1 C3435T and *ABCG*2 C421A polymorphisms in cases and controls are listed in Table 2 and Supplementary Figure 1. The observed genotype frequency among individuals in the control group was in agreement with Hardy–Weinberg equilibrium. There was no significant difference in the distribution of three genotypes (CC, CT, and TT) of *ABCB*1 C3435T between the breast cancer cases and healthy controls (*p*>0.05). No significant difference was detected in the distribution of C and T alleles between the breast cancer patients and healthy controls (*p*>0.05). Moreover, we did not find significant difference in the distribution of three genotypes (CC, CA, and AA) of *ABCG*1 C421A between the breast cancer cases and healthy controls (*p*>0.05). There were no significant difference in the distribution of C and A alleles between the breast cancer patients and healthy controls (*p*>0.05).

**Table 2:** Genotype and allele frequencies of *ABCB*1 C3435T and *ABCG*2 C421A polymorphisms in normal tissues of breast cancer patients and controls

	Variable	No. of cases	No. of controls^a^	P-value^b^	OR (95% CI)^c^
*ABCB*1 C3435T	Allele				
	C	122	120	-	-
	T	78	80	0.919	1.043(0.698-1.557)
	Genotype				
	CC	40	35	-	
	CT	42	50	0.353	1.361(0.738-2.508)
	TT	18	15	1	0.952(0.419-2.166)
	CT+TT	60	65	0.559	1.238(0.698-2.197)
*ABCG*2 C421A	Allele				
	C	135	133		
	A	65	67	0.915	1.046 (0.69-1.587)
	Genotype				
	CC	47	46	-	
	CA	41	41	1	1.022 (0.564-1.85)
	AA	12	13	1	1.107 (0.457-2.678)
	CA + AA	53	54	1	1.041 (0.597-1.815)

OR: odds ratio, CI: confidence interval

^a^ The observed genotype frequency among individuals in the control group was in agreement with Hardy–Weinberg equilibrium. (p^2^ + 2pq + q^2^)= 1; χ^2^ = 0.174, *p* = 0.677 for *ABCB*1 (C3435T); χ^2^ = 0.636, *p* = 0.425 for *ABCG*2 (C421A).

^b^ P values were calculated from two-sided chi-square tests for either genotype distribution or allele frequency

^c^ OR and 95 % CI values were calculated by unconditional logistic regression adjusted for age and menopausal state.

### Comparison of genotype distribution between breast cancer tissues and adjacent tissues

Overall genotype and allele frequencies for *ABCB*1 C3435T and *ABCG*2 C421A polymorphisms in cancer tissues and adjacent tissues are presented in Table 3. The observed genotype frequency among the para-carcinoma tissues was in agreement with Hardy–Weinberg equilibrium. There were no significant difference in the distribution of three genotypes (CC, CT, and TT) of *ABCB*1 C3435T between the carcinoma and para-carcinoma adjacent tissues (*p*>0.05). However, we found that 16% of carcinoma tissues had genetic mutations (Table 4). The mutation rates of CC, CT, and TT genotypes were 15.8%, 11.6%, and 26.3%, respectively. Although no significant difference was found in the distribution of three genotypes (CC, CA, and AA) of *ABCG2* C421A between the cancerous tissues and adjacent tissues (*p*>0.05), 17% mutation rate was detected in the cancerous tissues. The mutation rates of CC, CA, and AA genotypes were 1.9%, 43.3%, and 22.2%, respectively.

**Table 3:** Genotype and allele frequencies of *ABCB*1 C3435T and *ABCG*2 C421A polymorphisms in cancer tissues and adjacent tissues

	Variable	No. of cancer tissue	No. of adjacent tissue^a^	P-value^b^	OR (95% CI)^c^
ABCB1 C3435T	Allele				
	C	119	122	-	-
	T	81	78	0.838	0.939(0.629-1.402)
	Genotype				
	CC	38	40	-	-
	CT	43	42	0.876	0.928(0.502-1.716)
	TT	19	18	0.843	0.9(0.411-1.969)
	CT+TT	62	60	0.885	0.919(0.521-1.623)
ABCG2 C421A	Allele				
	C	134	135	-	-
	A	66	65	0.915	1.023 (0.674-1.553)
	Genotype				
	CC	52	47		
	CA	30	41	0.214	1.512 (0.818-2.795)
	AA	18	12	0.534	0.738 (0.322-1.692)
	CA+AA	48	53	0.572	1.222 (0.701-2.128)

OR: odds ratio, CI: confidence interval

^a^ The observed genotype frequency among cases in the adjacent tissues was in agreement with Hardy–Weinberg equilibrium. (p^2^ + 2pq + q^2^)= 1; χ^2^ = 1.375, *p* = 0.241 for *ABCB*1 (C3435T); χ^2^ = 0.429, *p* = 0.512 for *ABCG*2 (C421A).

^b^ P values were calculated from two-sided chi-square tests for either genotype distribution or allele frequency

^c^ OR and 95 % CI values were calculated by unconditional logistic regression adjusted for age and menopausal state.

**Table 4:** The mutation rates of *ABCB*1 C3435T and *ABCG*2 C421A in breast cancer patients

	Adjacent tissue	Cancer tissue		Mutation rate
*ABCB*1 C3435T	CC	CT	4	15.8%
		TT	2	
	CT	CC	1	11.6%
		TT	4	
	TT	CC	2	26.3%
		CT	3	
	Total		16	16%
*ABCG*2 C421A	CC	AA	1	1.9%
	CA	AA	9	43.3%
		CC	4	
	AA	CA	2	22.2%
		CC	2	
	Total		17	17%

### Relation between genotype distribution and clinicopathological characteristics

Clinicopathological features of the patients were distinguished according to *ABCB*1 C3435T and *ABCG*2 C421A genotypes and are shown in Table 5. Chi-square tests or Fisher’s exact test was used to assess the effect of the SNPs on the clinicopathological characteristics of the 100 breast cancer patients. We found that there was no significant correlation between genotype distribution of *ABCG*2 and age at diagnosis, menopausal state, age at menarche, histology, clinical stage, lymph node metastasis, Ki67 expression level, ER status, PR status, or HER2 status. We also investigated the effect of *ABCB*1 C3435T genotype distribution on the above clinicopathological features, and only clinical stage and Ki67 expression level were significantly associated with *ABCB*1 C3435T genotype. Moreover, we detected that the distribution frequency of the *ABCB*1 C3435T CC genotype was lower among the cases diagnosed with stage II than stage III (*p*= 0.018 and OR (95 % CI) 0.34 (0.146–0.793)). Based on our research, compared to patients with intermediate/high expression of Ki67, patients with low expression of Ki67 showed a significant reduction in mutation rate of ABCB1 C3435T (*p*< 0.001 and OR (95 % CI) 4.656 (1.922–11.279)).

**Table 5:** Correlation of clinical characteristics of *ABCB*1 C3435T and *ABCG*2 C421A polymorphisms in patients with breast cancer

Characteristics	*ABCB*1 C3435T				*ABCG*2 C421A			
	CC (No.)	CT+TT (No.)	*P*^a^	OR^b^ (95% CI)	CC (No.)	CA+AA (No.)	*P*^a^	OR^b^ (95% CI)
Age (year)								
<50	16	28	0.765	0.883 (0.391-1.996)	23	21	0.961	1.02 (0.463-2.248)
≥50	22	34			29	27		
Menopausal state								
Premenopausal	18	30	0.921	0.96 (0.428-2.155)	25	23	0.987	1.006 (0.459-2.207)
Postmenopausal	20	32			27	25		
Pathological type								
IDC	32	43	0.125	2.357 (0.845-6.572)	39	36	1	1 (0.404-2.474)
Others	6	19			13	12		
Clinical stage								
II	18	45	0.018	0.34^*^ (0.146-0.793)	35	28	0.41	1.471 (0.651-3.324)
III	20	17			17	20		
Lymph node metastssis								
Node-negative	16	32	0.412	0.682 (0.302-1.539)	23	25	0.548	0.73 (0.332-1.604)
Node-positive	22	30			29	23		
ER								
-	13	17	0.506	1.376 (0.575-3.292)	12	18	0.131	0.5 (0.209-1.194)
+	25	45			40	30		
PR								
-	15	25	1	0.965 (0.423-2.202)	18	22	0.309	0.626 (0.28-1.4)
+	23	37			34	26		
Her2								
-	25	35	0.405	1.484 (0.642-3.427)	30	30	0.685	0.818 (0.367-1.826)
+	13	27			22	18		
Ki67								
Low (<14%)	21	13	<0.001	4.656	14	20	0.12	0.516
Intermediate (14%-30%) and High (>30%)	17	49		(1.922-11.279)	38	28		(0.223-1.194)

OR: odds ratio, CI: confidence interval

^*^: *p*<0.05

^a^ P values were calculated from two-sided chi-square tests or Fisher’s exact test

^b^ OR and 95 % CI values were calculated by unconditional logistic regression adjusted for age and menopausal state.

### Association between *ABCB*1 and *ABCG*2 gene variants and patient survival

**Figure 1:**
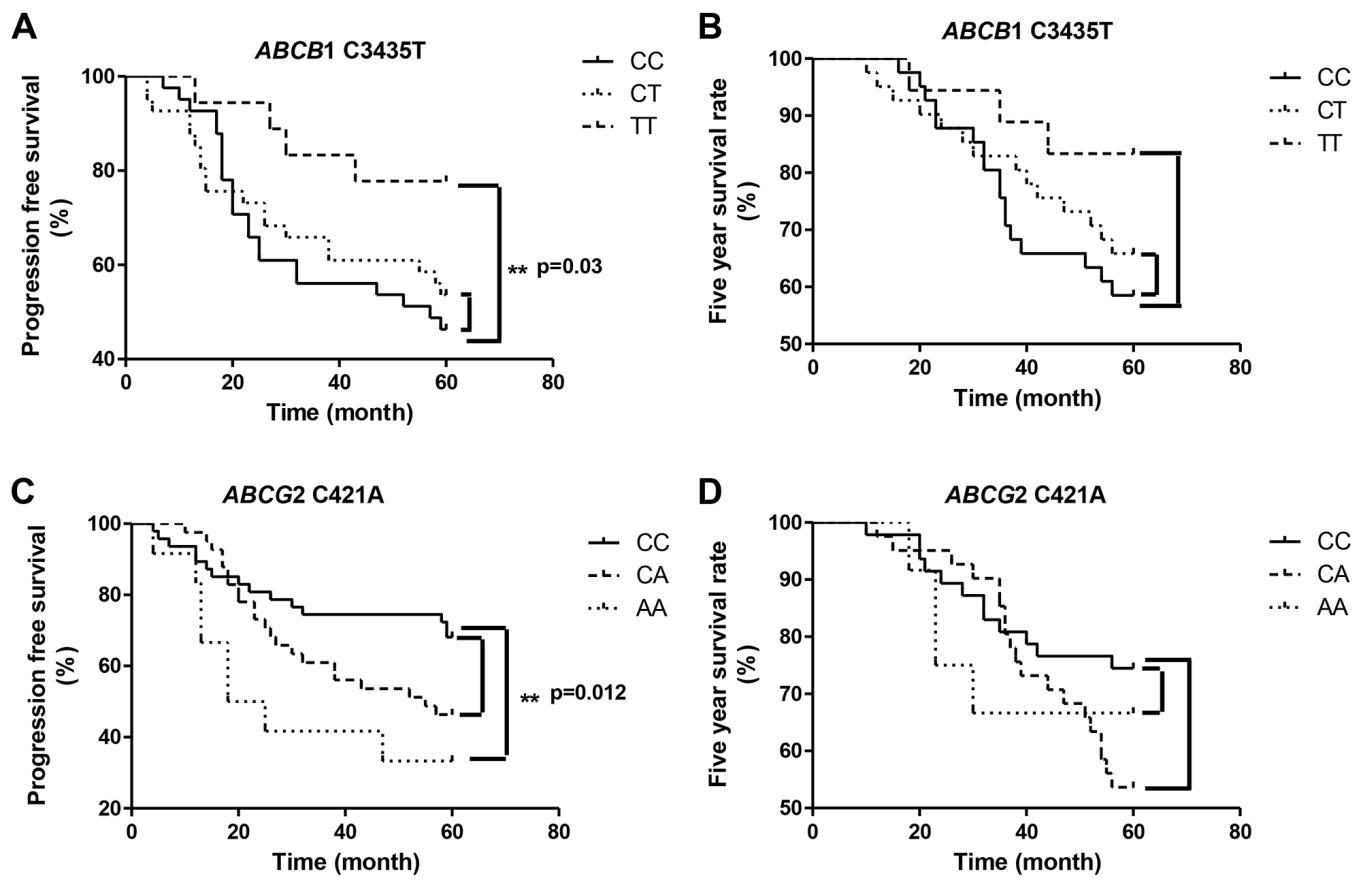
Association between *ABCB*1 and *ABCG*2 polymorphism and breast cancer patient survival **(A)** Association between *ABC*B1 C3435T polymorphism and progression-free survival. **(B)** Association between *ABCB*1 C3435T polymorphism and five year survival rate of the patients. **(C)** Association between *ABCG*2 C421A polymorphism and progression-free survival. **(D)** Association between *ABCG*2 C421A polymorphism and five year survival rate of the patients.

To investigate the association between *ABCB*1 C3435T and *ABCG*2 C421A gene polymorphisms and progression-free survival (PFS) and 5-year survival rate in breast carcinoma patients, the cases were followed up for 5 years. We observed that patients with TT genotype showed significantly longer PFS than patients harboring CC genotype in *ABCB*1 C3435T (p=0.03). There was no statistically significant difference in PFS between the patients with the CC genotype and those with the CT genotype in *ABCB*1 C3435T (p>0.05). Moreover, there was no significant association between the genotype of *ABCB*1 C3435T and the 5-year survival rate of breast cancer patients (Figure 1, p>0.05). We also detected that patients with CC genotype had significantly longer PFS than patients with 421AA genotype in *ABCG*2 (p=0.012). However, there was no significant difference in PFS between the patients with CC genotype and those harboring the CA genotype in *ABCG*2 C421A (p>0.05). There was no significant association between the polymorphism of *ABCG*2 C421A and the 5-year survival rate of breast cancer patients (Figure 1, p>0.05).

## DISCUSSION

Gene polymorphism plays a vital role in human phenotypic variability including cancer susceptibility and patient response to therapy. In recent decades, considerable progress has been made in the study of the polymorphism of tumor-resistance genes. *ABCB*1 and *ABCG*2 have been extensively studied as important resistance genes. Previous studies have shown that the polymorphisms of *ABCB*1 and *ABCG*2 can alter the mRNA expression levels and protein activity [11, 12, 21]. The C3435T polymorphism in *ABCB*1 is a synonymous mutation that can alter its mRNA expression level, protein activity, and substrate specificity [13-15]. The C421A polymorphism in *ABCG*2 can lead to a glutamine-to-lysine amino acid substitution, resulting in the decrease of its expression level and protein activity [22-24]. In this study, we examined the genotype frequencies of *ABCB*1 C3435T and *ABCG*2 C421A distribution in Chinese female breast cancer patients and healthy controls.

The frequencies of the CC, CT, TT genotype of C3435T in the breast cancer cases were 40%, 42%, and 18%, and in the healthy controls, the frequencies were 35%, 50%, and 15%, respectively. Our results suggested that there was no significant association between *ABCB*1 C3435T polymorphism and the breast cancer susceptibility in Chinese women (*p*>0.05). Tatari *et al.* also reported that *ABCB*1 C3435T polymorphism was not associated with breast cancer susceptibility in Iran [26]. Gervasini *et al.* found no association between *ABCB*1 C3435T polymorphism and risk of lung cancer [27]. In contrast, Siegsmund *et al.* reported that in renal cell carcinoma patients, the frequency of the exon 26 C3435T allele was significantly higher than the normal population [28]. The study of Jamroziak *et al.* in acute lymphoblastic leukemia showed that the *ABCB*1 3435TT genotype was associated with the occurrence of ALL. Wu *et al.* found that the frequency of the homozygous variant TT genotype of C3435T in breast carcinoma patients was significantly higher than in controls [29]. The difference in tumor type and patient ethnicity may contribute to these inconsistent findings. The frequencies of the CC, CA, and AA genotype of C421A in the breast carcinoma patients were 47%, 41%, and 12%, while 46%, 41%, and 13% in healthy controls, respectively. However, our findings suggested that *ABCG*2 C421A polymorphism was not associated with the susceptibility to breast cancer in Chinese women. A number of studies indicated that *ABCG*2 C421A polymorphism was not associated with the susceptibility to prostate cancer [30], colorectal cancer [31, 32]. In contrast, some studies suggested that *ABCG*2 C421A polymorphism may be useful as a biomarker for the prediction of susceptibility to diffuse large B-cell lymphoma [33], lymphoma [34], and nonpapillary renal cell carcinoma [35]. Previous studies have shown that the *ABCG*2 C421A polymorphism varies widely among different tumor types and populations. Nonetheless, research on the correlation of *ABCG*2 gene polymorphism and breast cancer susceptibility is lacking, which this study attempts to address in a Chinese Han population.

We also detected *ABCB*1 C3435T and *ABCG*2 C421A genotypes in the cancerous and normal tissues from breast cancer patients. Interestingly, we found that the cancer tissue of 16% breast carcinoma patients harbored gene mutations in the *ABCB*1 C3435T loci. The mutation rate of the CC, CT, and TT genotype was 15.8%, 11.6%, and 26.3% respectively. We also found that 17% of carcinoma cases had gene mutations in *ABCG*2 C421A loci. The mutation rate of the CC, CA, and AA genotype was 1.9%, 43.3%, and 22.2% respectively. Therefore, genotype analysis of cancer tissue cannot be replaced by detecting the peripheral blood or normal tissue of patients.

This study analyzed the correlation between the clinicopathological features of breast cancer patients and *ABCB*1 C3435T and *ABCG*2 C421A gene polymorphisms. The frequency of 3435CC genotype in patients with stage III was significantly higher than patients with stage II. Compared to patients with intermediate/high expression of Ki67, patients with low expression of Ki67 got a significant higher frequency of 3435CC genotype. Other clinicopathological features in this study were not significantly related to *ABCB*1 C3435T polymorphisms. Turgut *et al.* [36], Wu *et al.*[9] and Macías-Gómezdid *et al.* [37] reported similar results. The correlation between the clinicopathological features of breast carcinoma in our study, according to *ABCG*2 C421A polymorphism revealed no significant association at this level. Our results showed that no significant association between *ABCG*2 C421A polymorphisms and clinicopathological features (age at diagnosis, menopausal state, age at menarche histology, clinical stage, lymph node metastasis, Ki67 status, ER status, PR status, HER2 status) of breast cancer patients. Our results are in agreements with those from Korenaga *et al.* [35]. We speculate that other factors may obscure the relationship between *ABCG*2 C421A polymorphisms and clinicopathological characteristics.

All the patients in this study received surgical treatment and postoperative adjuvant chemotherapy. We followed up patients for five years, and assessed their progression-free survival and 5-year survival rates. Our results showed that breast carcinoma patients with *ABCB*1 3435TT genotype had significantly longer PFS than those with CC genotype. Our results were consistent with the studies of Madrid-Paredes *et al*[38] and Wu *et al*[9]. However, the studies of Cizmarikova *et al*[39] and Ji *et al*[40] are inconsistent with our findings. Potentially, the difference in pathological stage and treatment regimen may explain the inconsistent results. We also analyzed the 5-year survival rate of the patients, and found that there was no significant correlation with the polymorphisms of *ABCG*1 C34535T and *ABCG*2 C421A.

In conclusion, this study found that *ABCB*1 C3435T and *ABCG*2 C421A genotypes were not significantly correlation with the susceptibility to breast carcinoma in a Chinese Han population. However, these two loci had a higher rate of mutation in breast cancer tissue. *ABCB*1 3435TT and *ABCG*2 421CC genotypes were significantly correlated with longer PFS of the breast cancer patients. But the result of multivariate Cox regression analysis suggested that they cannot be used as predictors for the PFS of breast cancer patients (Table 6). Here, we suggest that in detecting breast cancer resistance-related genes, samples should be selected from cancer tissue, and not peripheral blood or normal tissue. Meanwhile, a multi-gene joint analysis will be better.

**Table 6:** Univariate and multivariate analysis of PFS in breast cancer patients

Variables	Univariate analyses		Multivariate analyses	
	Hazard ratio (95%CI)	*p*-value^a^	Hazard ratio^b^ (95%CI)	*p*-value^a^
Age	1.064(0.587-1.928)	0.838	-	-
Lymph node metastssis	0.852(0.473-1.535)	0.594	-	-
Pathological type	1.435(0.74-2.84)	0.286	-	-
Clinical stage	1.073(0.584-1.973)	0.281	-	-
Menopausal state	0.657 (0.342–1.324)	0.246	-	-
ER	2.136(1.103-4.136)	0.024^*^	1.787 (0.983-3.248)	0.057
PR	1.836(0.998-3.379)	0.051	-	-
HER2	0.666(0.362-1.226)	0.192	-	-
Ki67	0.999(0.54-1.847)	0.997		
*ABCB*1 C3435T	2.124 (1.035-4.358)	0.04^*^	1.785 (0.753-4.231)	0.188
*ABCG*2 C421A	2.039 (1.129-3.681)	0.018^*^	0.577 (0.312-1.067)	0.08

OR: odds ratio, CI: conWdence interval

^*^: *p*<0.05

^a^ P values were calculated from two-sided chi-square tests or Fisher’s exact test

^b^ OR and 95 % CI values were calculated by unconditional logistic regression adjusted for age and menopausal state.

## MATERIALS AND METHODS

### Study subjects

In this study, 100 breast cancer patients (female, median age: 50 years, range: 23–77 years) with incident breast carcinoma who were admitted to the 3201 hospital affiliated to Xi’an Jiao Tong University between 2010 and 2016 were enrolled. In addition 100 healthy control subjects (female, median age: 50 years, range: 20–75 years) were enrolled. All breast cancer patients underwent surgical treatment. Post-operative chemotherapy was based on a docetaxel and epirubicin regimen. Carcinoma and para-carcinoma tissues were collected from the patients and blood samples were collected from the healthy donors. Malignancy of the carcinoma tissues was confirmed by pathological analysis. The local ethics committee approved the research protocol for this study and all volunteers signed the study informed consent form.

### Date collection

Two clinicians collected clinical features and treatment outcomes from medical records and followed patients on a regular basis. Complete information about the treatment was obtained from all 100 breast carcinoma patients.

### DNA extraction

Paraffiwn-embedded tissue DNA extraction kit (TIANGEN, DP331, China) was used to extract DNA from carcinoma and para-carcinoma tissues. Blood genomic DNA extraction kit (TIANGEN, DP318, China) was used to extract DNA from blood samples. All protocols were in strict accordance with the manufacturers’ instructions.

### Single nucleotide polymorphism analysis

The SNPs in *ABCB*1 C3435T and *ABCG*2 C421A were detected by Taqman method using the primer sequences and probes shown in Table 7. Each PCR reaction mixture contained 2х Hotstart Fluo-PCR mix 10 μl, sense primer 0.5 μl (10 μM), anti- sense primer 0.5μl (10 μM), probe 0.8 μl (10 μM), template DNA 2 μl (20 ng/μl), PCR-grade water 6.2 μl. The amplification consisted of an initial denaturation step for 4 min at 95°C followed by 40 cycles of melting 95°C for 15 s, and annealing/extension at 60°C for 60 s. PCR reactions were carried out in a Roche, LightCycler480 Real-time PCR System.

**Table 7:** Primers and probes used for taqman assays

SNP	Primer	Probe
rs2231142	F:5’-ATGTTGTGATGGGCACTCTG-3’	P-A:TGCTGAGAACT**T**TAAGT
	R:5’-GTCATAGTTGTTGCAAGCCG-3’	P-C:TGCTGAGAACT**G**TAAGT
rs1045642	F:5’-CCTATGGAGACAACAGCCG-3’	P-T:CCTCAC**A**ATCTCTTC
	R:5’-ACTCGATGAAGGCATGTATGTT-3’	P-C:CTCAC**G**ATCTCTTC

### Immunohistochemical analysis

A standard protocol was used for the immunohistochemistry (IHC) of the samples that were detected as breast cancer by hematoxylin and eosin staining. Briefly, formalin fixed, paraffin embedding, paraffin-embedded specimens, dewaxing to water, antigen repair, serum blocking, primary antibody incubation (ER antibody, abcam, ab27595; PR antibody, abcam, ab32063; HER2 antibody, abcam, ab16901; Ki67 antibody, abcam, ab8191), secondary antibody incubation, coloration.

### Statistical analyses

All statistical analyses were carried out using Statistical Program for Social Sciences (SPSS) software 17.0 (SPSS Inc., USA). Hardy–Weinberg equilibrium and pairwise haplotype frequencies were estimated using the Hardy–Weinberg calculator and CubeX tools respectively, both provided by the Online Encyclopedia for Genetic Epidemiology studies. Statistical significance was set at *p* < 0.05 for all tests, and all tests were two-sided. Chi-square (Pearson’s χ^2^ test) or Fisher’s exact test was used to determine the differences in distributions of demographic, epidemiologic, and clinical variables between the two groups. Survival analysis was performed by Kaplan–Meier method and compared by log-rank test. Factors with significant influence on univariate analysis were further analyzed by multivariate Cox regression analysis. The minimum level of significance was established at *p*<0.05.

### Compliance with ethical standards

The study was approved by the ethics committee of 3201 hospital affiliated to Xi’an Jiaotong University.

## SUPPLEMENTARY MATERIALS FIGURE


