# Supplementary material for: ABCB1 3435TT and ABCG2 421CC genotypes were significantly associated with longer progression-free survival in Chinese breast cancer patients
Source: Oncotarget. 2017 Oct 31;8(67):111041–52. doi: 10.18632/oncotarget.22201 (PMC5762303; doi:10.18632/oncotarget.22201)
Supplement: Supplementary file 1 [file oncotarget-08-111041-s001.pdf]

ABCB1 3435TT and *ABCG2* 421CC genotypes were significantly associated with longer progression-free survival in Chinese breast cancer patients

SUPPLEMENTARY MATERIALS

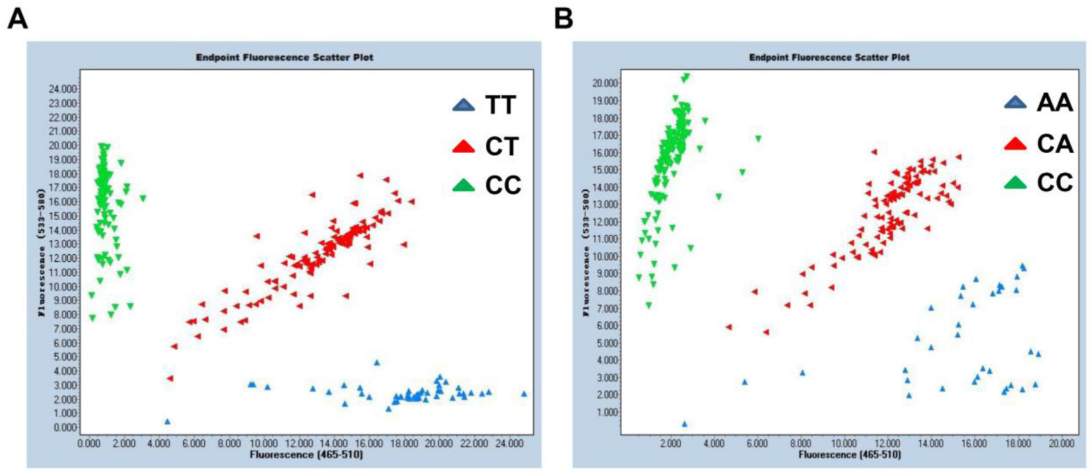

Supplementary Figure 1: *ABCB*1 **and** *ABCG*2 gene polymorphisms detection results **(A)** ABCB1 C3435T, **(B)** ABCG2 C421A.
